# A Robust Random Forest-Based Approach for Heart Rate Monitoring Using Photoplethysmography Signal Contaminated by Intense Motion Artifacts

**DOI:** 10.3390/s17020385

**Published:** 2017-02-16

**Authors:** Yalan Ye, Wenwen He, Yunfei Cheng, Wenxia Huang, Zhilin Zhang

**Affiliations:** 1 School of Computer Science and Engineering, University of Electronic Science and Technology of China, Chengdu 611731, China; yalanye@uestc.edu.cn (Y.Y.); hwwuestc@gmail.com (W.H.); yunfeicheng@hotmail.com (Y.C.); 2 West China Hospital of Sichuan University, Chengdu 610041, China; 3 Samsung Research America—Dallas, Richardson, TX 75082, USA

**Keywords:** heart rate (HR), photoplethysmography (PPG), motion artifacts (MA), random forest

## Abstract

The estimation of heart rate (HR) based on wearable devices is of interest in fitness. Photoplethysmography (PPG) is a promising approach to estimate HR due to low cost; however, it is easily corrupted by motion artifacts (MA). In this work, a robust approach based on random forest is proposed for accurately estimating HR from the photoplethysmography signal contaminated by intense motion artifacts, consisting of two stages. Stage 1 proposes a hybrid method to effectively remove MA with a low computation complexity, where two MA removal algorithms are combined by an accurate binary decision algorithm whose aim is to decide whether or not to adopt the second MA removal algorithm. Stage 2 proposes a random forest-based spectral peak-tracking algorithm, whose aim is to locate the spectral peak corresponding to HR, formulating the problem of spectral peak tracking into a pattern classification problem. Experiments on the PPG datasets including 22 subjects used in the 2015 IEEE Signal Processing Cup showed that the proposed approach achieved the average absolute error of 1.65 beats per minute (BPM) on the 22 PPG datasets. Compared to state-of-the-art approaches, the proposed approach has better accuracy and robustness to intense motion artifacts, indicating its potential use in wearable sensors for health monitoring and fitness tracking.

## 1. Introduction

Heart rate (HR) estimation based on wearable devices is of vital importance due to its useful features in controlling the training load or monitoring physiologic conditions during daily activities. Photoplethysmography (PPG) [1,2,3,4,5] is a popular technique due to its simpler hardware implementation and lower cost than the conventional electrocardiography (ECG) method. However, PPG is susceptible to motion artifacts (MA), which can become very strong during subjects’ intense physical exercise, hindering the estimation of HR using PPG.

To eliminate MA effectively in the presence of strong MA, various methods, such as independent component analysis (ICA) [6,7], adaptive filtering [8,9] and empirical mode decomposition (EMD) [10], have been investigated. Besides, one type of hybrid method was proposed to improve the denoising performance, such as [11,12], which combined two MA removal algorithms. This type of combination may cause high computational complexity, since the use of the second algorithm may cause unnecessary computation when noise has been reduced effectively by the first algorithm. Recently, another type of hybrid method was proposed to improve the denoising performance with a low computational complexity, such as [13,14], where two algorithms were combined by a binary decision algorithm based on the correlation coefficient (CC) whose aim was to decide whether or not to adopt the second algorithm. However, CC can only detect if there exists a linear relationship between the denoised PPG signal and the reference signal. A correlation coefficient close to zero simply indicates that two signals are not linearly related, but they still may be highly correlated in a nonlinear sense, indicating that CC would not work well at this point, and the denoising performance may be affected.

Simultaneously, to estimate HR from seriously contaminated PPG, one kind of algorithm, called the spectral peak tracking algorithm, was proposed to locate the spectral peak of HR in the spectrum of the denoised PPG signal. In [15], a heuristic rules-based spectral peak tracking algorithm, which relied on the frequency harmonic relation of HR, was presented. Then, various heuristic algorithms have been proposed sequentially, such as [12,16,17,18,19]. However, these algorithms rely on a number of heuristic rules, where the adjustment of parameters is arbitrary, and a dozen parameters are required to be specified, increasing the risk of poor generalization and robustness on the unseen data. In [20], instead of using a heuristic algorithm, a Bayesian decision-based algorithm was exploited to track the spectral peak of HR. However, this algorithm requires a prior distribution for all unknown parameters. If selecting an improper prior, the performance of the algorithm would be severely affected.

In this paper, a robust HR estimation approach based on random forest is proposed to estimate HR from the PPG signal contaminated by intense motion artifacts, including a hybrid MA removal method (Stage 1) and a random forest-based spectral peak tracking algorithm (Stage 2). Stage 1 aims at eliminating MA and getting a cleansed PPG signal. Next, in the spectrum (calculated by periodogram) of the cleansed PPG signal, Stage 2 exploits the random forest-based spectral peak tracking algorithm to identify the spectral peak of HR. The main contributions of this paper are as follows:

- The proposed hybrid MA removal method can not only improve the denoising performance, but also hold a low computational complexity by random forest-based binary decision algorithm, which combines two MA removal algorithms. Compared with the correlation coefficient-based binary decision algorithm that can only detect the linear relationship [13,14], the proposed binary decision algorithm can detect not only the linear relationship by using the correlation coefficient as one feature, but also the nonlinear relationship by using many other effective features, ensuring an accurate decision result and thus improving the denoising performance considerably with a low computational complexity.
- The spectral peak tracking problem is formulated into a pattern classification task, and the random forest-based algorithm can locate the spectral peak corresponding to HR with a better generalization and robustness. Most existing heuristic tracking algorithms set rules artificially and adjust the parameters arbitrarily, resulting in a poor robustness on a more challenging dataset. In contrast, the random forest-based algorithm can set more formalized rules and can adjust the parameters by an intelligent classifier, achieving a better robustness and generalization. Hence, the proposed spectral peak tracking algorithm can be more suitable for wearable devices.

## 2. Stage 1: Motion Artifacts Removal

In Stage 1, the proposed binary decision algorithm combines two MA removal algorithms: second-order Volterra adaptive noise cancellation (ANC) and singular spectrum analysis (SSA). First, we adopt nonlinear adaptive filtering to suppress strong noise. The Volterra filter is very useful for nonlinear system, since it can be computed using traditional signal processing algorithms in the same way as linear models. After second-order Volterra ANC, the denoised PPG signal $\hat{s}$ is obtained. Second, the random forest-based binary decision is used to decide whether another the MA removal algorithm should be used to further remove MA by deciding that MA in $\hat{s}$ is Strong or NotStrong. Finally, if the result is Strong, SSA [21,22], which is another effective methodology for removing MA, would be further used. After SSA, a more cleansed PPG signal ${\hat{s}}_{recon}$ can be obtained. Otherwise, if the decision result is NotStrong, SSA would not be used, avoiding increasing unnecessary workload, and then, ${\hat{s}}_{recon}=\hat{s}$.

**Figure 1:**
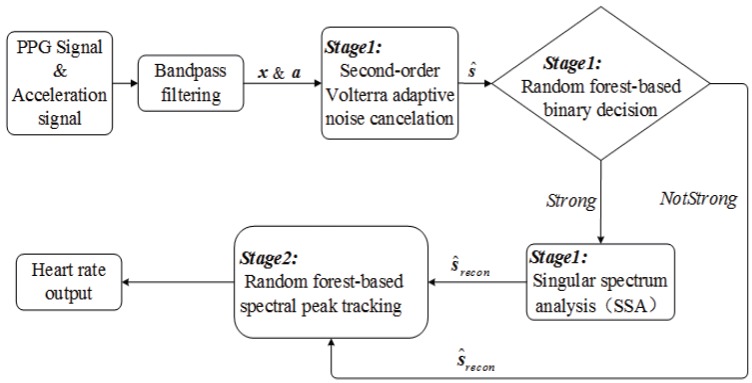
The flowchart of our proposed HR estimation approach.

**Figure 2:**
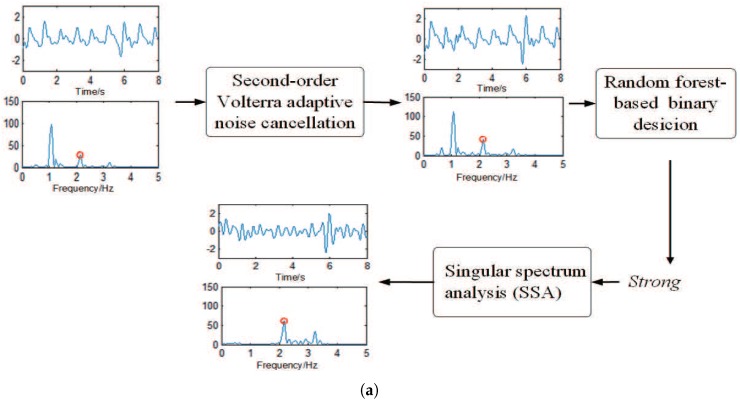

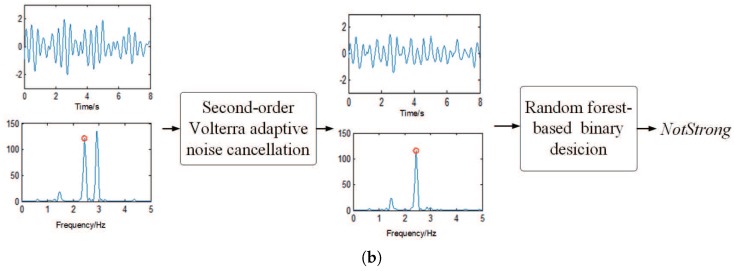
Two examples showing the process of removing MA. In (**a**,**b**), the waveform in the above layer is in the time domain, and the below layer is the corresponding spectrum. The red cycle in the spectrum represents the spectral peak corresponding to HR, which is obtained by simultaneous ECG. In (**a**), the MA in the denoised signal are determined as Strong, and it shows that a more accurate HR estimation can be obtained by further exploiting singular spectrum analysis (SSA). In example (**b**), the decision result is NotStrong, indicating that SSA should not be used to avoid increasing unnecessary workload. (**b**) shows that the spectral peak of HR still can become dominant by the first MA-removal algorithm without the use of SSA.

Figure 1 shows the flowchart of the proposed HR estimation approach, from which we can also see the flowchart of Stage 1. Then, to describe the process of removing MA vividly, Figure 2 gives two examples: in (a), the signal in one time window is classified as Strong, and it shows that the spectral peak associated with HR can only become dominant by the combination of second-order Volterra ANC and SSA; in other words, a more accurate HR estimation can be obtained by further exploiting SSA; in (b), the signal in another time window is determined as NotStrong, which shows that the spectral peak associated with HR can also become dominant without SSA. In other words, MA in the PPG signal that have been denoised by the first MA removal algorithm are not very strong, and there is no need to further use SSA.

Before our proposed HR estimation approach starts, as in [15], all of the raw signals would be band-pass filtered from 0.4 Hz to 5 Hz, which is the frequency band we are interested in. In this paper, a time window of *T* seconds was sliding on the raw signal with an incremental step of $M_{0}$ seconds (generally $M_{0}\leq T/2$).

### 2.1. Second-Order Volterra Adaptive Noise Cancellation

The aim of adaptive noise cancellation (ANC) is to obtain the denoised PPG signal by subtracting the estimated undesired noise (MA) using the second-order Volterra filter algorithm. For convenience, we consolidated the tri-acceleration signals into one channel of signal a by calculating the norm of the triaxial vector at every sampling point. Since the acceleration signal a and the undesired motion artifacts (MA) n have the same source, motion [9], the acceleration signal a is used as the reference signal in this paper. Since the signal measured from the PPG sensor is contaminated by strong MA, the measured PPG signal x is a combination of the desired pulse signal $s_{desire}$ and the motion artifacts n:

(1) $$x\left(k\right)=s_{desire}\left(k\right)+n\left(k\right);k=0,1,\ldots ,M-1,$$

where *M* is the length of the time window and x(k), $s_{desire}\left(k\right)$ and n(k) are the *k*-th element of x, $s_{desire}$ and n, respectively.

In this step, we use a truncated Volterra series expansion of second order to reform the reference signal and, thus, get the input signal $\hat{a}\left(i\right)$:

(2) $$\begin{array}{c} \hat{a}\left(i\right)=[a\left(i\right),a\left(i-1\right),\ldots ,a\left(i-N\right),a^{2}\left(i\right),\\ \;\;\;\;\;\;a(i)a(i-1),\ldots ,a(i)a(i-N),\ldots ,\\ \;\;\;\;\;\;a\left(i-N\right)a\left(i-N+1\right),a^{2}\left(i-N\right){]}^{T}, \end{array}$$

where i=0,1,…,k, a(i) is the element in a and *N* is the length of a.

The recursive least square algorithm is adopted to update filter coefficient w(k) used for extracting motion information, since it has a fast convergence speed and an excellent performance [23]. The optimal vector w(k) can be given by:

(3) $$w(k)={\left[\sum_{i=0}^{k}\lambda^{k-i}\hat{a}\left(i\right){\hat{a}}^{T}\left(i\right)\right]}^{-1}\sum_{i=0}^{k}\lambda^{k-i}\hat{a}\left(i\right)x\left(i\right),$$

where *λ* is the forgetting factor and x(i) is the *i*-th element of x. Then, the estimated pulse $\hat{s}\left(k\right)$ can be extracted by subtracting the estimated MA $\hat{n}\left(k\right)$ from the x(k) as follows:

(4) $$\hat{s}\left(k\right)=x\left(k\right)-\hat{n}\left(k\right)=x\left(k\right)-w{\left(k\right)}^{T}\hat{a}\left(k\right),$$

where $\hat{s}\left(k\right)$ is the *k*-th element of denoised PPG signal $\hat{s}$.

### 2.2. Random Forest-Based Binary Decision

The aim of this step is to detect if MA in the denoised PPG signal $\hat{s}$ are Strong or NotStrong. One commonly-used algorithm is random forest, which is a classifier consisting of a collection of tree-structured classifier [24]. Since random forest can exhibit a substantial performance improvement over many tree-based algorithms, it is selected as the classifier in our experiments. In our work, the ten-fold cross-validation method is used to evaluate the generalization performance of the classifier of the first stage.

#### 2.2.1. Random Forest-Based Classifier Training

Segment extraction for a training set: In this part, a set of segments is extracted to provide the ground truth of a training set. That is, extract the segments on behalf of Strong and NotStrong. The extraction rule is: in the spectrum of $\hat{s}$ obtained by the periodogram, if the HR value calculated by the frequency location index of the peak with the max amplitude is very close to the heart rate provided by ECG signal, MA in $\hat{s}$ of the current time window would be regarded as NotStrong; otherwise, it would be regarded as Strong.

Feature vector extraction: For each extracted segment, we extract not only common statistical features in time domain and frequency domain, but also the features in the time-frequency (wavelet) domain. In contrast to the Fourier transform (which is related to the frequency domain), the basis functions used in the wavelet transform (which is related to the wavelet domain) are temporally localized [25]. In other words, the features in the wavelet domain can yield a potentially more revealing picture of the temporal localization of a signal’s spectral components [26] that is not a simple combination of time and frequency information. Thus, the above property provides a particularly rich description of non-stationary signals, which often have a nonstationary frequency composition and burst-like temporal structure [26]. In fact, the PPG signal may contain non-stationary or transitory characteristics that are difficult to capture only by Fourier spectrum during subjects’ intense exercises. In this step, discrete wavelet transform (DWT) is used to decompose the PPG signal into frequency sub-bands where the statistical features of wavelet coefficients are calculated. The features in the time domain, frequency domain and time-frequency (wavelet) domain are all extracted for distinguishing between clean and corrupted PPG signal, introduced as follows.

- Time domain: the energy of the denoised PPG signal $\hat{s}$ would be selected as a feature;
- Frequency domain: (1) Firstly for the spectrum (calculated by periodogram) of the clean PPG signal, it contains few frequency components (a significant fundamental peak and several harmonic peaks). However, the spectrum of corrupted PPG signal is very messy. Therefore, the number of significant peaks is selected as a feature, where significant peak means that the amplitude of the peak is larger than a threshold $\delta_{1}$ of the maximum amplitude ($\delta_{1}=30\%$ in our experiments). (2) Then, the mean and kurtosis of the frequency spectrum of $\hat{s}$ are selected as the features. (3) Furthermore, the correlation coefficients between the spectrum of $\hat{s}$ and the raw PPG signal and the correlation coefficients between the spectrum of $\hat{s}$ and the acceleration signal are used as features. For example, for a clean PPG signal, the value of the correlation coefficients is very small, but for a corrupted signal, the value is large.
- Wavelet domain: Using wavelet transform, the denoised PPG signal $\hat{s}$ can be decomposed into a number of sub-band signals. (1) The energy of each of these sub-band signals is selected as a feature. (2) Then, the mean and standard deviation of these sub-band signals are selected as features. Specifically, the signal is decomposed into the fifth level using the mother wavelet of the Daubechies wavelet of order four (db4).

Then these statistical features would be used as an input (which is also called the feature vector) of the training set with two discrete outputs (which are also called class labels): Strong or NotStrong.

Random forest-based classifier training: The training set consisting of feature vectors and the corresponding class labels would be exploited to train the random forest-based classifier to obtain the classifier parameters for predicting.

#### 2.2.2. Binary Decision Using the Trained Classifier

After the classifier has been well trained, the parameters obtained by the previous step would be used to classify the PPG signal $\hat{s}$ in the current time window to one of the categories: Strong (MA in $\hat{s}$ are very strong) or NotStrong (MA in $\hat{s}$ are not very strong). If $\hat{s}$ is classified as NotStrong, the proposed algorithm would proceed directly to the next stage of spectral peak tracking, and ${\hat{s}}_{recon}=\hat{s}$ where ${\hat{s}}_{recon}$ is the cleansed PPG signal used in Stage 2. If $\hat{s}$ is classified as Strong, the proposed algorithm would proceed to the next step (SSA) to further remove MA.

### 2.3. Singular Spectrum Analysis

Singular spectrum analysis (SSA) aims to decompose the original series (namely the denoised PPG signal $\hat{s}$) into a small number of time series, so that each sub-series can be identified as either a trend, periodic or noise [27]. The time sub-series corresponding to noise (MA) can be identified with the aid of the acceleration signals [28]. The steps to eliminate MA by SSA are as follows.

- The periodogram is first used to get the spectrum of acceleration signals a. In the spectrum, we determine the dominant frequencies with an amplitude larger than a threshold $\delta_{2}$ ($\delta_{2}=50\%$ in our experiments) of the maximum amplitude. Denote by $L_{acc}$ the set of location indexes of selected dominant frequencies in the spectrum.
- Then, SSA is exploited to decompose the denoised PPG signal $\hat{s}$ which is the output of second-order Volterra ANC, into some time series [21,22].
- For each time series, if its dominant frequency has location indexes in $L_{acc}$, it would be regarded as the time series associated with MA [28]. Finally, the cleansed PPG signal ${\hat{s}}_{recon}$ can be obtained by summing the remained time series without the series corresponding to MA.

## 3. Stage 2: Random Forest-Based Spectral Peak Tracking

The aim of this stage is to locate the spectral peak associated with HR in the spectrum (calculated by periodogram) of ${\hat{s}}_{recon}$. It is mentioned that random forest [24] is exploited again, but compared with Stage 1, it is quite a different algorithm with quite a different aim. The flowchart of this stage is shown in Figure 1. In the following descriptions, some variables would be predefined first, and then, the random forest-based spectral peak tracking algorithm would be described in detail.

Before beginning, some variables are defined in the spectrum (calculated by periodogram) of ${\hat{s}}_{recon}$:

- $L_{prev}$ is the frequency location index of HR estimated in the previous time window.
- $L_{Range1}=\left[L_{prev}-\Delta_{s},\cdots ,L_{prev}+\Delta_{s}\right]$, where $L_{Range1}$ is the range of fundamental frequency of HR, and $\Delta_{s}$ is a small positive integer ($\Delta_{s}=2$ in our experiments).
- $L_{Range2}=\left[2\left(L_{prev}-\Delta_{s}-1\right)+1,\cdots ,2\left(L_{prev}+\Delta_{s}-1\right)+1\right]$, where $L_{Range2}$ is the range of first-order harmonic frequency of HR, and $\Delta_{s}$ is a small positive integer.
- $L_{i}^{0}\left(i=1,2\right)$ represents the frequency location indexes of two dominant peaks in $L_{Range1}$, and $L_{i}^{1}\left(i=1,2\right)$ is from $L_{Range2}$. In this stage, dominant peak denotes the spectral peak that has the dominant frequencies with an amplitude larger than a threshold $\delta_{2}$ (mentioned in the part of the introduction of SSA) of the maximum amplitude.
- Loc denotes the finally selected frequency location index of the spectral peak of HR at this stage.

### 3.1. Random Forest-Based Spectral Peak Tracking

The aim of this step is to classify the spectrum of ${\hat{s}}_{recon}$ in the current time window into: Class 1 (the first class label of the classifier), Class 2 (the second class label) or Class 3 (the third class label). Class 1 mainly means that there exists a harmonic pair in the spectrum of ${\hat{s}}_{recon}$. Class 2 mainly means that there is no harmonic pair, but the spectral peak associated with HR has been found. Class 3 indicates that the spectral peak associated with HR has not been found, but it just depends on the spectral peak of HR of the previous time window. For the three possible states of the spectrum, the spectrum peak corresponding to HR can be found out and denoted by $L_{candi}^{i}\left(i=1,2,3\right)$. In other words, three different spectrum states (Class 1, Class 2 and Class 3) that the classifier aims to classify correspond to a candidate peak set ($L_{candi}^{1}$, $L_{candi}^{2}$ and $L_{candi}^{3}$). Then, when estimating HR, if the result of the classifier is Class *l* (l=1, 2 and 3), $L_{candi}^{l}$ would finally become the final frequency location index of the spectral peak associated with HR. $L_{candi}^{1}$, $L_{candi}^{2}$ and $L_{candi}^{3}$ are defined as follows,

(5) $$L_{candi}^{1}=\left\{\begin{array}{cc} L_{i}^{0} & \left(L_{i}^{0},L_{j}^{1}\right)\left(i,j\in \left\{1,2\right\}\right)\\ L_{prev}\pm \sigma_{1} & otherwise \end{array}\right.$$

where $\left(L_{i}^{0},L_{j}^{1}\right)\left(i,j\in \left\{1,2\right\}\right)$ means that there exists a peak-pair $\left(L_{i}^{0},L_{j}^{1}\right)$ with a harmonic relation, $L_{prev}\pm \sigma_{1}$ means that $L_{prev}$ would be plus $\sigma_{1}$ or minus $\sigma_{1}$ according to the trend of $L_{i}^{0}$ and $\sigma_{1}$ is small positive ($\sigma_{1}=2$ in our experiments). Then, the principle for selecting the second one is:

(6) $$L_{candi}^{2}=\left\{\begin{array}{cc} L_{closest} & \left|L_{closest}-L_{prev}\right|\leq \sigma_{2}\\ L_{prev}\pm \sigma_{2} & otherwise \end{array}\right.$$

where $L_{closest}$ is the value (chosen from the set of $\left\{L_{1}^{0},L_{2}^{0},\frac{L_{1}^{1}-1}{2}+1,\frac{L_{2}^{1}-1}{2}+1\right\}$) closest to $L_{prev}$, the setting of $L_{prev}\pm \sigma_{2}$ is the same as $L_{prev}\pm \sigma_{1}$ and $\sigma_{2}$ is small positive ($\sigma_{2}=2$ in our experiments). Finally, the principle for selecting the third one is:

(7) $$L_{candi}^{3}=L_{prev}$$

where $L_{prev}$ is the third candidate peak, since in many cases, the spectral peak associated with HR keeps its location unchanged in two successive time windows.

#### 3.1.1. Random Forest-Based Classifier Training

**Figure 3:**
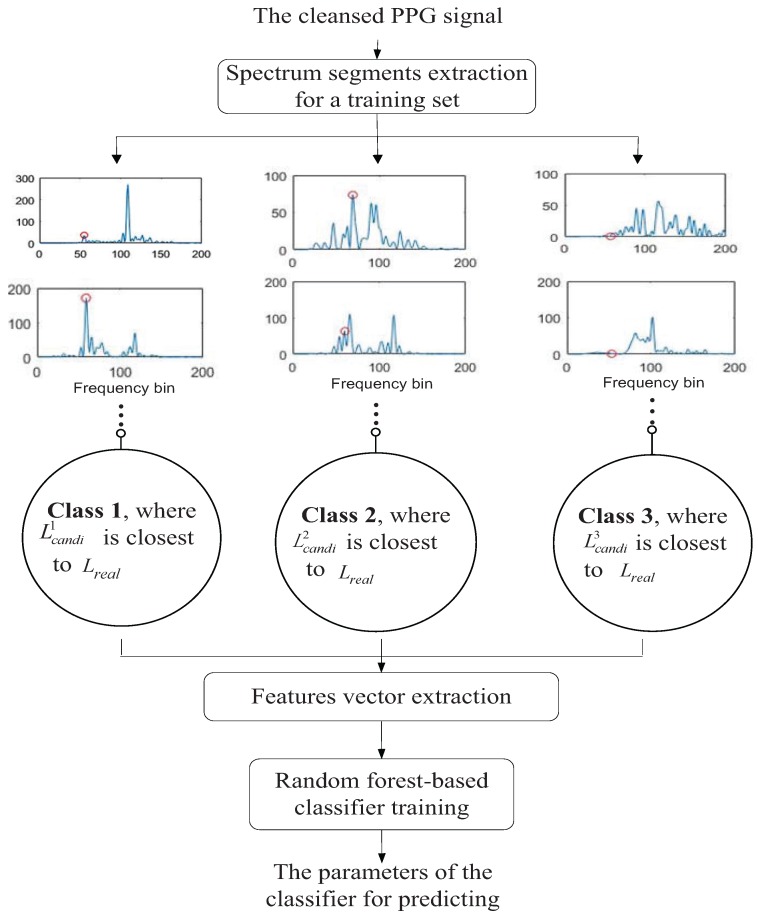
The training process of the random forest-based classifier in Stage 2.

To better understand random forest-based spectral peak tracking, the process of training the random forest-based classifier is described in detail. Figure 3 illustrates the process of classifier training. In this part, the ten-fold cross-validation method is used again to evaluate the generalization performance of the classifier of the second stage.

Spectrum segments extraction for a training set: Similar to the random forest-based binary decision algorithm introduced above, a set of spectrum segments is extracted for a training set. Here, one segment means one spectrum state of ${\hat{s}}_{recon}$ in one time window. Denote by $L_{real}$ the frequency location index converted from real heart rate value (from the ECG signal) through Equation (9). Note that the extracting principle is according to the distance between three candidate peaks ($L_{candi}^{1}$, $L_{candi}^{2}$ and $L_{candi}^{3}$) and $L_{real}$. For example, in the spectrum of ${\hat{s}}_{recon}$ in one time window, $L_{candi}^{1}$, $L_{candi}^{2}$ and $L_{candi}^{3}$ are all calculated out. If $L_{candi}^{1}$ is closest to $L_{real}$, this segment (time window) would be marked as Class 1. Similarly, if $L_{candi}^{2}$ is the closest, the segment would be marked as Class 2, or if $L_{candi}^{2}$ is the closest, it would be marked as Class 3.

Feature vector extraction: For each extracted segment, a number of features should be extracted to be fed to classifier. The descriptions of the features are as follows.

- Extract the number of dominant spectral peaks in the time window of $L_{Range1}$ and $L_{Range2}$, respectively. The reason is that for Class 1, the signal is relatively clean; thus, the number is less. However, for Class 3, the signal is relatively not clean; thus, the number is larger.
- Extract the energy of a, since signal a can indirectly reflect the state of the signal.
- Extract the correlation coefficient between ${\hat{s}}_{recon}$ and a and the correlation coefficient between the spectrum of ${\hat{s}}_{recon}$ and the spectrum of a. The smaller the correlation coefficient, the more clean the signal, then it is more likely to be Class 1.
- Extract the mean value, skewness and kurtosis of ${\hat{s}}_{recon}$. These statistical properties can capture the characteristics of the signal, such as the concentration trend of the signal.
- Extract a feature indicating the presence or absence of the peak-pair $\left(L_{i}^{0},L_{j}^{1}\right)$. If exists, the value of the feature is marked as Number 1, which indicates that Class 1 has a greater chance; if not, it is marked as Number 0, meaning that Class 2 and Class 3 have greater possibility.

The above features would form the input (called the feature vector) of the training set with three outputs (called class labels): Class 1, Class 2 and Class 3.

Random forest-based classifier training: After the training set including the feature vectors and the corresponding class labels is collected, it would be fed into the classifier. After training random forest-based classifier, the parameters for predicting can be obtained.

#### 3.1.2. Spectral Peak Tracking Using the Trained Classifier

In this step, the goal is to locate HR according to the parameters obtained by training random forest-based classifier of the previous step. If the class label of the classifier when detecting HR in the current time window is Class l(l=1,2, or 3), then:

(8) $$Loc=L_{candi}^{l}.$$

Note that after the location associated with HR Loc is found, it can be transformed to the HR value by the following equation:

(9) $$HR=\frac{Loc-1}{N}f_{s}\times 60,$$

where $f_{s}$ denotes the sampling rate and *N* denotes the number of frequency bins [15].

## 4. Datasets and Performance Metrics

### 4.1. Datasets

The datasets exploited for evaluating the proposed HR estimation approach are provided by the 2015 IEEE Signal Processing Cup, which were also used in [14,15]. The datasets consist of a two-channel PPG signal recorded from the wrist by two pulse oximeters with green LEDs (wavelength: 515 nm), the tri-axis acceleration signals recorded from the wrist by a tri-axis accelerometer and an ECG signal recorded from the chest using wet ECG sensors. The datasets include 22 recordings collected from 18–58-year-old subjects performing various physical exercises, like running and rehabilitation exercises. The ground truth of HR in each time window is calculated from the simultaneous ECG signal, and it is now available in the datasets for performance evaluation.

The first 12 of the 22 recordings were used in the evaluation of TROIKA [15]. They were recorded during subjects’ walking, jogging and running on a treadmill with speeds of 1–2 km/h for 0.5 min, 6–8 km/h for 1 min, 12–15 km/h for 1 min, 6–8 km/h for 1 min, 12–15 km/h for 1 min and 1–2 km/h for 0.5 min. For the remaining 10 of the 22 recordings, each of the subjects performed many actions, including various forearm and upper arm exercises (e.g., shake hands, stretch, push, and so on, which are common in arm rehabilitation exercise), running, jump and push-up, where MA are more strong than the first 12 recordings.

### 4.2. Metrics

In this paper, three indexes are used to evaluate the performance of our proposed HR estimation approach.

Firstly, the average absolute error (AAE) is defined as:

(10) $$AAE=\frac{1}{W}\sum_{i=1}^{W}|BPM_{est}\left(i\right)-BPM_{true}\left(i\right)|,$$

where BPM is beats per minute, $BPM_{true}\left(i\right)$ represents the ground truth of HR in the *i*-th time window, $BPM_{est}\left(i\right)$ denotes the estimated HR values and *W* is the total number of time windows.

Then, the Bland–Altman plot is used to verify the agreement between the ground truth of HR and the estimated HR values. Here the limit of agreement (LOA) expressed by $\left[\mu -1.96\sigma ,\mu +1.96\sigma \right]$ is also calculated, where *μ* is the average difference and *σ* is the standard deviation.

The last index is the Pearson correlation coefficient between the ground truth and the estimates.

Note that the smaller AAE and the absolute value of *μ* are, the better estimation performance of the approach is. Additionally, a high Pearson correlation coefficient indicates a good HR estimation.

## 5. Experimental Results

### 5.1. Experimental Setting

In the simulation, the PPG datasets were used to test the performance of our proposed HR estimation approach. As mentioned above, a time window of *T* seconds was sliding on the raw signal with an incremental step of $M_{0}$ seconds (generally $M_{0}\leq T/2$), where *T* was set to 8 s and $M_{0}$ was set to 2 s. Since the sample frequency $f_{s}$ of all signals was set to 125 Hz in our experiment, we set the number of frequency bins N=4096. Furthermore, the length of the time window (M=125×8) was 1000. In the second-order Volterra ANC algorithm, the time delay (between the corrupted PPG signal and the acceleration data) was set to 0.08 s, which can make the acceleration signal a highly correlated with the noise n and help the filtering algorithm work well [29]. For the classifier used in Stage 1, using the mentioned features, the classifier can achieve good performance. Specifically, the testing accuracy of the classifier of this step reaches 96.76%; the sensitivity is 94%; and the specificity is 97.72%; where three indexes (the testing accuracy, sensitivity and specificity) are used to evaluate the performance of the classifier. High values of the three indexes mean good performance for the classifier. For the other classifier used in Stage 2, the testing accuracy of the classifier reaches 98.63%. In this work, some of the latest HR estimation approaches with good performance were chosen for comparison [13,14,15,20,30,31].

### 5.2. Results and Discussions

Table 1 presents the average absolute error (AAE) for each subject’s recording and gives an overall average AAE. The average AAE of the proposed HR estimation approach across the first 12 of 22 recordings was 1.23 ± 0.80 (mean ± standard deviation) BPM, for the remaining 10 was 2.16 ± 2.10 BPM and for all 22 recordings was 1.65 ± 1.56. Note that the proposed approach achieved an accurate estimation not only on the the first 12 recordings, but also on the more challenging 10 recordings, showing that the proposed approach not only can obtain accurate performance, but also has good robustness.

In Table 1, there are also two groups of the latest HR estimation approaches chosen for comparison: those that put forward spectral peak tracking algorithms (including TROIKA [15], JOSS [30], SpaMA [31] and SPECTRAP [20]) and those that put forward hybrid MA removal methods with the correlation coefficient-based decision algorithm (including CC [13] and CNAFSD [14]).

In the first group, the first three approaches [15,30,31] were all based on heuristic rules, and the last one [20] was based on the Bayesian decision rule. From the results, we can see that heuristic algorithms achieved a good performance on the 12 recordings, but a poor performance under the more challenging 10 recordings. The result indicates that heuristic algorithms are not robust, and the reason is that heuristic algorithms always rely on experience-based rules and parameters. These rules and parameters are mainly obtained based on the existing datasets; however, the specified rules and parameters may not work well when dealing with the more challenging 10 recordings recorded when MA are stronger than the 12 recordings. Instead of using the heuristic algorithm, SPECTRAP [20] exploited the Bayesian decision based on the prior distribution to track the spectral peak. In fact, in many cases, prior knowledge is either vague or non-existent [32]. If selecting an improper prior, the performance of the algorithm would be severely affected.

In the second group, CC [13] and CNAFSD [14] both exploited the correlation coefficient (CC)-based binary decision. According to the results, the proposed random forest-based binary decision algorithm was better than the CC-based decision algorithm. The reason is that correlation coefficient is not robust, which can only detect if there exists a linear relationship (between the denoised PPG signal and the acceleration signal), but cannot detect a nonlinear relationship, resulting in poor decision accuracy and, thus, poor HR estimation accuracy. Compared with CC, the proposed random forest-based binary decision algorithm can exploit many features, including the correlation coefficient, to effectively monitor if there exists very strong MA, obtaining more accurate binary decision and, thus, achieving a better HR estimation.

**Figure 4:**
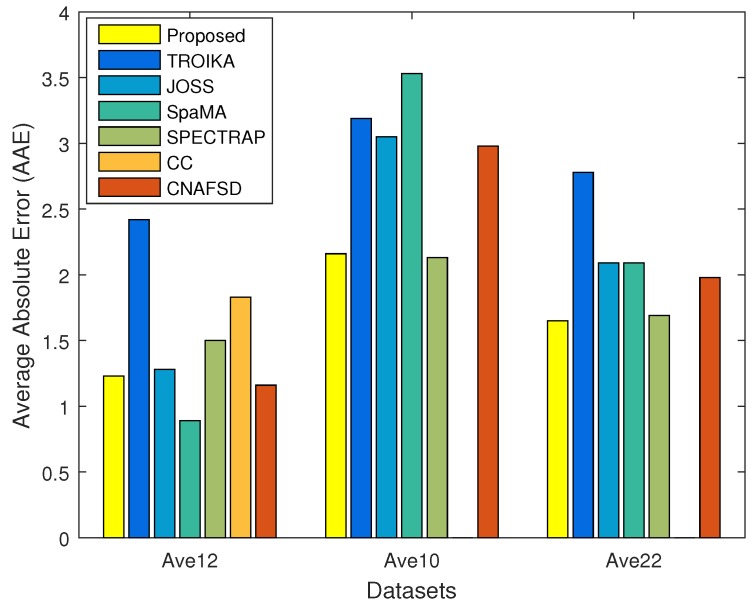
The bar graph of HR estimation results for the approaches listed in Table 1 (the proposed HR estimation approach, TROIKA [15], JOSS [30], SpaMA [31], SPECTRAP [20], CC [13] and CNAFSD [14]) in terms of average AAEs on the first 12 recording, the remaining 10 challenging recordings and all 22 recordings.

To better show the average AAEs of several HR estimation approaches listed in Table 1, Figure 4 gives a bar graph of HR estimation results for the proposed approach and two groups of the latest HR estimation approaches chosen for comparison in terms of average AAEs on the first 12 recordings, the 10 challenging recordings and the 22 recordings. From this figure, we can see that, compared with the two groups of the latest HR estimation approaches [13,14,15,20,30,31], the proposed approach can achieve good performance not only on the the first 12 recordings, but also on the 10 challenging recordings, and thus, achieve good performance on the 22 recordings. This figure indicates that the proposed approach not only can obtain accurate performance, but also has good robustness on challenging recordings.

**Figure 5:**
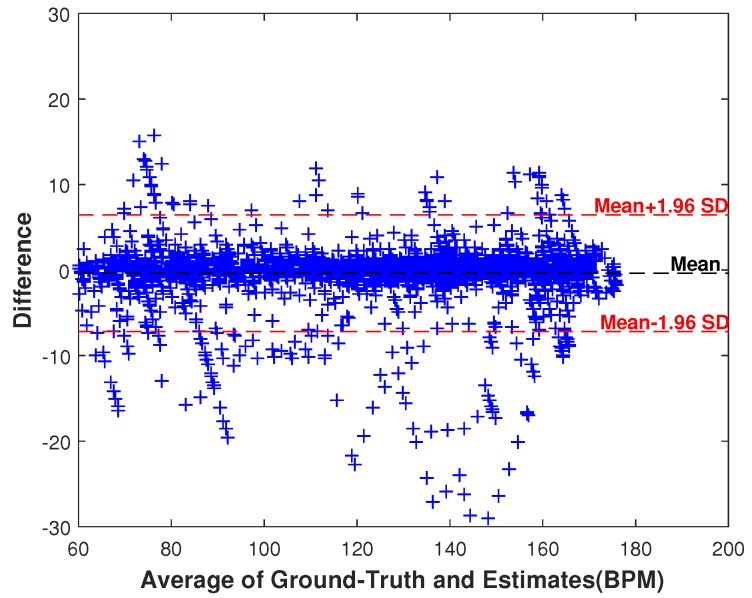
The Bland–Altman plot of the estimates of our proposed approach on the 22 datasets. The limit of agreement (LOA) was [−7.18, 6.46] BPM (standard deviation *σ* = 3.48 (BPM).

To better see the performance of our proposed HR estimation approach for the 22 subjects, Figure 5 gives the Bland–Altman plot, which is used to verify agreement between the ground truth of HR and the estimated HR values. In this figure, the limit of agreement (LOA) expressed by $\left[\mu -1.96\sigma ,\mu +1.96\sigma \right]$ was [−7.18, 6.46] BPM (the absolute value of mean *μ* = 0.36 BPM, standard deviation *σ* = 3.48 BPM). From Figure 5, we can see that the absolute value of mean *μ* is very small, indicating good estimation performance of the proposed HR estimation approach.

**Figure 6:**
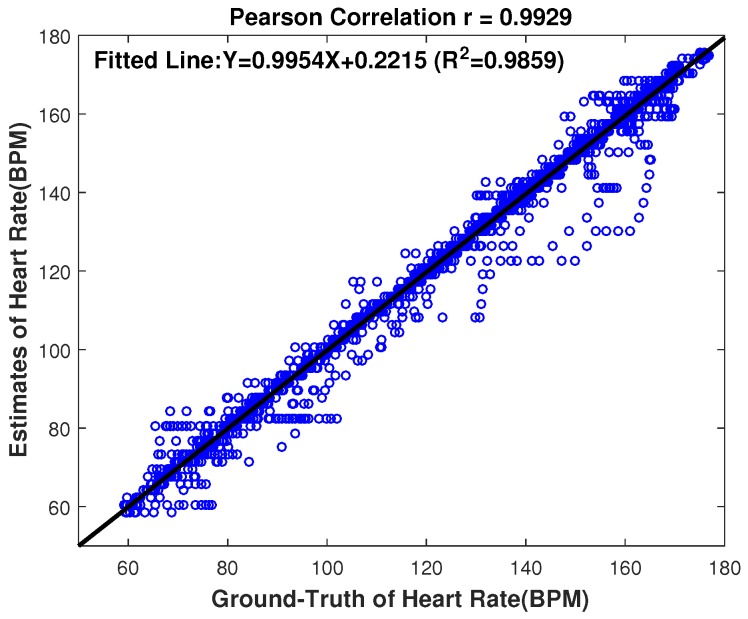
Scatter plot on the 22 datasets between the ground truth and the estimates of our proposed approach. The fitted line was y=0.9954x−0.2215; the $R^{2}$ value was 0.9859; the Pearson correlation correlation was 0.9929.

Furthermore, to better see the performance of the proposed approach, Figure 6 gives the scatter plot between the ground truth and the estimates, where the fitted line was y=0.9954x−0.2215 (*x* is the ground truth heart rate value and *y* is the associated estimate); the $R^{2}$ value, which is the measure of goodness of fit, was 0.9859; and Pearson correlation coefficient was 0.9929. From Figure 6, we can see that the estimated HR values are quite close to the ground truth as we expected, and the Pearson correlation coefficient is very high, indicating a good HR estimation using the proposed HR estimation approach.

**Figure 7:**
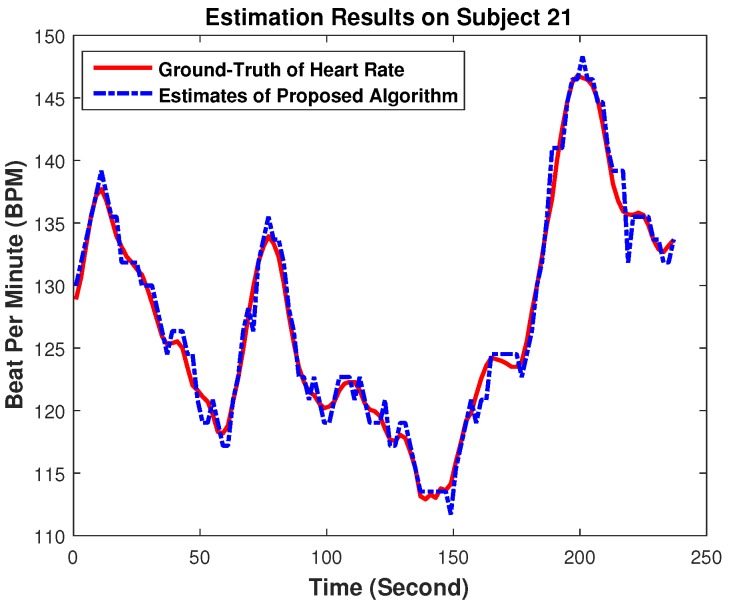
Estimation results on recordings of Subject 21 of 22 recordings. The HR traces of our proposed approach were plotted, and this was compared to the ground truth, which was recorded simultaneously from ECG.

To further show the performance visually, Figure 7 gives the estimation results on the recordings of Subject 21 (randomly chosen). From the figure, we can see that the estimated HR values of our proposed HR estimation approach are very close to the ground truth, indicating the high accuracy of our performance again.

## 6. Conclusions

In this work, a robust HR estimation approach using random forest, based on the PPG signal contaminated by intense motion artifacts, is proposed for fitness tracking by wearable devices, such as smart watches and smart wristbands. The proposed approach consists of the stage of motion artifacts removal (second-order Volterra adaptive noise cancellation, random forest-based binary decision, singular spectrum analysis) and the stage of random forest-based spectral peak tracking. It can remove MA effectively with a low computational complexity and locate the spectral peak corresponding to HR with a better robustness and generalization, thus achieving a high accuracy and robustness for HR estimation. Experimental results on datasets including 22 subjects showed excellent performance of the proposed HR estimation approach, indicating its potential use in wearable devices for health monitoring and fitness tracking.

## Figures and Tables

**Table 1:** The HR estimation results in terms of AAE on the 22 PPG datasets. Average absolute error (AAE).

Subject	Proposed	TROIKA [15]	JOSS [30]	SpaMA [31]	SPECTRAP [20]	CC [13]	CNAFSD [14]
Sub.1	1.61	2.87	1.33	1.23	1.18	2.06	1.66
Sub.2	1.39	2.75	1.75	1.59	2.42	3.59	1.56
Sub.3	0.73	1.91	1.47	0.57	0.86	0.92	0.65
Sub.4	1.48	2.25	1.48	0.44	1.38	1.54	1.48
Sub.5	0.77	1.69	0.69	0.47	0.92	0.97	0.77
Sub.6	1.34	3.16	1.32	0.61	1.37	1.64	1.12
Sub.7	0.59	1.72	0.71	0.54	1.53	2.25	0.72
Sub.8	0.63	1.83	0.56	0.40	0.64	0.63	0.91
Sub.9	0.57	1.58	0.49	0.40	0.60	0.62	0.42
Sub.10	3.50	4.00	3.81	2.63	3.65	4.62	2.35
Sub.11	1.07	1.96	0.78	0.64	0.92	1.30	1.45
Sub.12	1.04	3.33	1.04	1.20	1.25	1.80	0.78
Sub.13	5.24	6.63	8.07	3.41	4.891	-	7.71
Sub.14	1.12	1.94	1.61	7.29	1.58	-	1.62
Sub.15	1.31	1.35	3.10	2.73	1.83	-	3.10
Sub.16	6.81	7.82	7.00	3.18	3.05	-	7.00
Sub.17	1.76	2.46	2.99	3.01	1.62	-	2.99
Sub.18	1.26	1.73	1.67	4.46	1.24	-	1.67
Sub.19	1.62	3.33	2.80	3.58	2.04	-	2.45
Sub.20	0.91	3.41	1.88	1.94	2.49	-	1.81
Sub.21	0.92	2.68	0.92	2.56	1.16	-	0.92
Sub.22	0.64	0.51	0.49	3.12	0.66	-	0.49
Ave12 (mean ± SD)	1.23 ± 0.80	2.42 ± 0.78	1.28 ± 0.90	0.89 ± 0.60	1.50 ± 0.86	1.83 ± 1.21	1.16 ± 0.55
Ave 10 (mean ± SD)	2.16 ± 2.10	3.19 ± 2.32	3.05 ± 2.52	3.53 ± 1.48	2.13 ± 1.21	-	2.98 ± 2.45
Ave 22 (mean ± SD)	**1.65 ± 1.56**	2.78 ± 1.67	2.09 ± 1.99	2.09 ± 1.73	1.69 ± 1.06	-	1.98 ± 1.90
